# The policy statement of the American academy of pediatrics – children as hematopoietic stem cell donors – a proposal of modifications for application in the UK

**DOI:** 10.1186/1472-6939-14-43

**Published:** 2013-10-31

**Authors:** Tak Kwong Chan, George Lim Tipoe

**Affiliations:** 1 Anatomy, University of Hong Kong, L1-59, Laboratory Block, 21 Sassoon Road, Hong Kong, Hong Kong; 2 Department of Anatomy, University of Hong Kong, L1-59, Laboratory Block, 21 Sassoon Road, Hong Kong, Hong Kong

**Keywords:** Policy, Haematopoietic stem cell transplant, Ethical concerns, Donor siblings

## Abstract

**Background:**

With a view to addressing the moral concerns about the use of donor siblings, the Policy Statement of the American Academy of Pediatrics - Children as Hematopoietic Stem Cell Donors (the Policy) has laid out the criteria upon which tissue harvest from a minor would be permissible.

**Discussion:**

Although tissue harvest serves the best interests of recipient siblings, parents are also obliged to act in the best interests of the donor sibling in the UK. Tissue harvest should proceed if and only if it serves the best interests of both the donor and recipient. Parents should be forbidden, and they are by UK law, to consent to tissue harvest unless there are substantial benefits for an incompetent minor that can outweigh the potential harm. There is no basis to subject a minor to the medical risks of tissue harvest if the recipient sibling can wait without significant risks of complications until the donor becomes Gillick competent. We also argue that the Policy fails to take into account recent advances in haematopoietic transplantation from haploidentical donors or related tissue-matched donors.

**Summary:**

Unless a recipient sibling will suffer from serious complications or die without the transplantation and no other medically equivalent donors are available, there is no moral or legal basis to violate the donor sibling’s right to bodily integrity. Accordingly, we propose that the Policy should be modified in order to fully satisfy the legal requirements for application in the UK and other commonwealth jurisdictions with similar statute laws protecting minors.

## Background

In conjunction with preimplantation genetic diagnosis (PGD), assisted reproduction can be used to avoid inheritable diseases. After successful in-vitro fertilization, PGD can identify whether any of the embryos is affected by a genetic condition. Parents now can even request selection of embryos on the basis of tissue type in order to create a child to cure an existing sick child. In response to the moral concerns about the use of paediatric haematopoietic cell donor siblings (donor siblings), the Policy Statement of the American Academy of Pediatrics – Children as Hematopoietic Stem Cell Donors (the Policy) suggests that tissue harvest should only proceed upon satisfaction of all the following five criteria:

1) there is no medically equivalent histo-compatible adult relative who is willing and able to donate;

2) there is a strong personal and emotionally positive relationship between the donor and recipient;

3) there is some likelihood that the recipient will benefit from the transplantation;

4) the clinical, emotional, and psychosocial risks to the donor are minimized and are reasonable in relation to the benefits expected to accrue to the donor and to the recipient; and

5) parental permission and, where appropriate, child assent have been obtained’ [1].

Except that the fourth criterion requires the physician to minimise the risks of medical procedures by, e.g. avoiding central venous catheter insertion and reducing psychological risk by proper communication skills and the fifth requires that child assent should be obtained where appropriate, the remaining criteria seem to merely state the facts common to most cases of tissue harvest rather than regulating the process. We contend that the Policy fails to sufficiently protect the interests of minor donors. In this article we would like to review the prevailing UK law, discuss the ethical relevance, and propose modifications of the Policy for application in the UK and other commonwealth jurisdictions with similar statute laws.

## Discussion

### The legal principles

One would not dispute that tissue harvest serves the best interests of recipient siblings. But we are also obliged to look at the welfare of the donor sibling. In Birmingham City Council v H (a minor), Balcombe LJ said the question of welfare of both children should be approached 'without giving one priority over the other’ [2]. In the UK, any persons beyond the age of 16 can consent to medical treatment. Where a child is below the age of 16, as established in the case of Gillick, a child could consent if he or she fully understands the medical treatment that is proposed (i.e. Gillick competent) [3]. In case a donor sibling has not yet achieved sufficient understanding and intelligence to fully understand the proposed transplantation, the Children Act 1989 requires that a parent must act in the best interests of the donor [4]. In Re C (HIV Test), the parents of a baby born to a HIV positive mother opposed the testing of the child for HIV. Wilson J stated that the views of the parents were important factors in the decision but that "the baby had rights of her own recognised in national and international law, the baby’s welfare was paramount, and in the baby’s interests the test should take place" [5]. The assessment of best interests is a balance of the benefits and risks of transplantation for the donor. The procedures need not be risk free but parents should only consent to tissue harvest if the possible benefits for the donor outweigh the potential harm. Furthermore, if a person under the age of 18 refuses to consent to a treatment, it is possible for their parents to overrule their decision. However, this right can be exercised only for the welfare of a child, and in the case of tissue transplantation, the Children Act 1989 empowers the court to intervene if parents fail to act in the best interests of the donor sibling [4,6].

### The best interests of donor siblings

#### Well-documented risks

The most ethically sound basis to oppose tissue harvest is that it imposes risks of bodily injuries associated with the medical procedures of tissue harvest. The risks depend on the source of cells namely bone marrow, peripheral blood and umbilical cord blood, each with their own benefits and risks. It is known that harvest by umbilical cord blood carries no medical risks except in case of modified delivery. Therefore our analysis below focuses on bone marrow and peripheral blood transplantation only. Minor complications like pain at collection sites, fatigue and pain may occur in a small proportion of donors (6%-20%). Major and life-threatening complications following marrow donation like anesthesia-related events, injuries to bones, sacroiliac joint and sciatic nerve were estimated to occur in 0.1%-0.3% of cases [7]. Major risks of peripheral blood transplantation are mainly associated with sedation, general anaesthesia and central venous catheter insertion, estimated to be about 1.1% of cases [8]. In light of one’s inviolable right to bodily integrity, minors should not be exposed to the medical risks unless there are well-established benefits of a significant magnitude that they can accrue from tissue harvest.

#### Potential benefits

The possible benefits for donor siblings are merely psychosocial. In an American case Curran v. Bosze, Calvo J said that 'the psychological benefit is grounded firmly in the fact that the donor and the recipient are known to each other in a family. There may be a psychological benefit to the child from donating bone marrow to a sibling’ [9]. We consider that tissue harvest can have short-term and long-term psychosocial effects for donor siblings. When presented with the idea of donation, younger donors were found to focus more on the immediate pain and the frightening experience of the procedure [10], whereas older children might feel pressure from family members or medical staff to become a donor [11]. MacLeod and others found that about a third of the siblings donors thought that doctors and family members limited their opportunity to say no, making them perceive they had a 'forced no choice’ [10]. And donor siblings reported higher levels of anxiety and lower self-esteem than non-donor siblings [12], which may be attributed to the lack of choice in the decision to donate. It follows that a donor sibling is more likely to have short-term psychosocial burdens rather than benefits.

Long term positive effects for the donor siblings include greater sensitivity to the needs of others [13], enhanced self-sufficiency and independence [14], increased family closeness [15], and most important of all, the possible companion of a healthy recipient sibling. On the other hand, negative emotions were reported when their recipient siblings developed long-lasting complications from the procedure. Furthermore, anger, guilt, and blame were common amongst donor siblings with unsuccessful transplant [10]. While the Policy cited the findings of three studies that all donor siblings agreed that the psychosocial benefits and harm of serving as a donor outweighed the physical harms, Macleod, Wieners and others found that only 66.6% and 67% of donor siblings reported a predominantly positive psychosocial experience [10,16]. Importantly, all reporting predominantly positive psychosocial experience had successful transplant and only donors with unsuccessful transplant reported a predominantly negative experience in Macleod’s study [10]. Chang and others also showed that donors for recipients who die remain significantly more depressed than donors for recipients who survive 6 months after donation [17]. As the psychosocial experience is highly associated with the outcome for the recipient, we contend that the long-term benefits for donor siblings more likely derive from the survival of the recipient sibling rather than from process of tissue harvest itself.

#### Balance of benefits and risks

Whereas the advantages of tissue harvest for donor siblings are long-term and merely psychosocial, the other side of balance sheet consists of short-term psychological burden and life-threatening physical harm. One should bear in mind that the notion of benefits and risks are both relative concepts. To consider the potential gains or losses for donor siblings, we ought to consider what would likely happen to the donors and recipients without tissue harvest. It cannot be said that tissue harvest can offer any significant long-term benefits for the donor sibling if other available source of transplant can offer a similar chance of success or if the sick sibling has a high chance of survival without serious complications even without transplantation. Those who do not agree may argue that a donor sibling may in the future relish and take pride in being a donor and therefore still want to donate even if other donors are available.

Parents may legitimately consider this factor when making decisions for incompetent minors. However, the sense of pride may not materialize if the grown-up donor sibling does not accept the filial or social responsibility to save the recipient sibling, is disinterested in altruistic donation, or does not relish in living in the shadows of the recipient sibling. It is too speculative to suggest that an autonomous individual would necessarily prize the honour of 'a donor and saviour’ above all things if there are other suitable donors willing to donate or if the recipient is not in the most dire need of transplantation. As the Policy permits transplantation only if there is no medically equivalent histo-compatible adult relative who is willing and able to donate, it is beyond dispute that a moral agent should not subject a minor to the risks of tissue harvest if there are other equally good alternatives. It implies that the sense of pride alone is not an overriding factor in assessing the benefits and for a minor donor. In short, the only circumstance where tissue harvest would likely serve the best interests of a minor donor is that without the proposed transplantation taking place, the sick sibling will have a high chance of dying or suffering from serious complications.

### The ethical basis of the legal approach

We hold that the above legal approach is in line with the Beauchamp and Childress’ principled-based approach of ethical analysis [18]. As one does not have a legitimate claim to life to such an extent that it unjustly causes harm to another person, [19] the principles of beneficence, non-maleficence, and justice demand that parents should consent to tissue harvest only if it serves the best interests of both the donor and recipient.

#### Huge benefits v minimal risks

In response, one may argue that the requirement that a sick sibling will suffer serious complications or die without the proposed transplantation is too high a bar. However, as we have illustrated in the legal analysis above, unless this requirement is satisfied, the donor sibling is unlikely to benefit from the tissue harvest. Month said 'I do not believe that a day of minor discomfort and an extremely small risk of anesthesia outweigh a lifetime without a healthy older brother or sister and the years of joy it potentially brings’ [20]. It is tempting but erroneous to balance the benefits for the sick sibling rather than those for the donor against the medical risks of transplantation for the donor. The rationale that minimal bodily invasion with low risks is a justification is also seriously flawed as it is not the degree of the risks of a medical procedure but the bodily invasion itself that matters. The right to have intact bodily integrity is a matter of the right to privacy irrespective of the degree of invasion [21]. If such invasion is not allowed for adults without their consent, there is no basis to allow this for children [22]. So the benefits must be so substantial for the donor that it relates to the survival of the sick sibling with a view to justifying the risk of transplantation.

#### Conflict of interests

One may then question the validity of the claim that transplantation can proceed only if it also serves the best interests of the donor sibling. Beauchamp and Childress gave little guidance regarding the approach of resolving conflicts of interests between two persons. But it is generally accepted that when there are conflicts between two principles, we should first minimise the effects of violation. As the UK court has previously held, pursuant to prevailing family law principles, it was bound to make the least detrimental choice [23].

Transplantation will not benefit the donor sibling, as we contend above, when there is another source of medically equivalent tissue from a mentally competent adult or when the sick sibling will not die or suffer from serious complications without the transplantation. In the former case, to minimize the effect of violation of autonomy, we should accept donation from a competent adult who agrees to donate rather than harvest tissue from a minor who cannot consent. In the latter, as the sick sibling can still survive in an acceptably healthy condition even without transplantation, the least detrimental choice is to wait until the donor can understand the process of transplantation (i.e. Gillick competent) and decide whether he would like to agree to altruistic donation.

#### Familial duties

Lastly, one may argue that there is a special duty to rescue a sick sibling as a result of a family relationship. Dwyer and Vig held that a donation is justifiable even if it does not benefit the donor sibling, so long as there is 'a moral match between the relationship and the risks to the donor relative to the benefits to the recipient’ [24]. But putting the entire situation in the context of a family hardly advances an argument in favour of tissue harvest in case the best interests of the donor are not served. Members within a family usually have different relationships with one another complicated with different interests, rights and obligations. While a parent might feel obliged to donate to a child, a sibling may not necessarily feel the same.

We submit that it is only fair to impose an obligation to a member of any units or organisations under one of the two following conditions. First, it is considered that the interests of the unit or organisation prevail over the interests of any individual member. Nonetheless, the infringement of a member’s rights as a result of such an imposition of duty must be the least possible infringement without which the primary goal commensurate with the resulting infringement cannot be achieved. In the case of tissue harvest from a donor sibling, this condition will be satisfied only if other sources of medically equivalent tissue from a mentally competent adult are not available. Further, it will not be otherwise proportionate to violate the donor’s bodily integrity unless the recipient sibling has a high chance of dying or suffering from serious complications without transplantation. Second, an individual agrees, either by word or by conduct, to abide by a set of norms or regulations that impose such an obligation while entitling him to certain privileges. In the circumstance, it cannot be legitimate to impose parents’ view of familial obligations on a child who is not yet able to appreciate his rights and obligations in the family [21]. In fact, if the first condition is satisfied, transplantation will also serve the best interests of the donor sibling and there will be no need to invoke the concept of familial duties to justify tissue harvest.

### Availability of other donors

In light of foregoing discussion, to determine whether tissue harvest can be legally and ethically permitted, one should assess first whether the recipient sibling would have a high chance of dying or suffering from serious complications without a transplantation. If the answer is affirmative, then one should see if donation from any mentally competent donors can offer a similar chance of successful transplantation.

In the past, tissue-matched related donors were considered to be the best sources of donation. But now, many drawbacks of unrelated or haplo-identical transplants such as graft failure and significant graft-versus-host diseases have been overcome due to the development of new extensive T-cell depletion techniques with mega dose stem cell administration over the last two decades. New approaches such as alloreactive T cell depletion and post-transplant cell therapy have also improved immune reconstitution in tissue-matched unrelated or haplo-identical transplants. Using haplo-identical donors are now considered to be equally safe and effective transplant strategies as tissue-matched donors for advanced leukemia [25]. Leung and others showed that the five year survival rates of recipient siblings with high risk leukemia are promising regardless of donor types (68% for donor siblings, 74% for unrelated donors and 77% for haplo-identical donors), and that the risks of death from graft-versus-host diseases at five years is not significantly associated with the types of donors (6.6% for donor siblings, 7.0% for unrelated donors, and 3.4% for haplo-identical donors) [26]. Saber and others also showed that for adult patients with acute myeloblastic leukemia, transplantation from tissue-matched unrelated donors and tissue matched related donors do not differ in terms of 3-year survival, relapse and cumulative incidences of chronic graft-versus-host diseases three years after transplantation [27]. Therefore, a search for competent family members, local and even international bone marrow donor registry should be undertaken before parents are permitted to consent to tissue harvest from donor siblings for recipients suffering from advanced lymphoma or leukemia. For other diseases, Sodani and others reported that haplo-identical transplant in thalassemia has 93% chance of survival and only 7% mortality using new conditioning regimen [28]. Bolaños-Meade and others showed that the use of haplo-identical transplant for patients with sickle cell disease is highly effective and safe despite the fact that graft failure still remains an obstacle [29].

### Proposed amendments of the policy

It is already well established that the use of haplo-identical transplant is equally safe and effective for advanced lymphoma or leukemia, whereas studies are still underway to evaluate the results with non-malignant diseases like aplastic anemia, lupus and sickle cell diseases. The above literature search by no means suggests that haplo-identical transplant can replace tissue-matched transplant but, given the success of new regimens of transplantation, doctors should look beyond the availability of tissue-matched related donors for certain types of diseases. Parents and doctors should make sure, if the medical conditions of the sick sibling permit a search, that no other sources of equally or similarly effective transplant are available before subjecting a minor to the physical risks and short-term psychological harm of tissue harvest.

In the circumstances, we propose that the following should replace the original first, third, and fifth criteria in the Policy:

1. If the medical conditions of the recipient permit a search, there is no medically equivalent haplo-identical/histo-compatible related adult or histo-compatible unrelated adult who is willing and able to donate;

3. The recipient sibling will have a high chance of dying or suffering from serious complications without transplantation before the donor sibling becomes Gillick competent;

5. Parental permission is obtained if the donor sibling is not Gillick competent.

## Summary

The parental choice to harvest tissues from donor siblings reflects the immense 'emotive desire to do something to help the recipient sibling’ [30]. However, donor siblings may not necessarily reap any benefits in light of the risks of serious injuries arising from bone marrow or peripheral blood transplantation. Fulfilling the criteria specified in the Policy of the Academy alone is not sufficient to justify tissue harvest from minors. Unless a recipient sibling will likely suffer from serious complications or die without the transplantation and no tissue-matched unrelated donors or related haploidentical donors are available, there is no moral or legal basis to violate the donor sibling’s right to bodily integrity. In short, to apply the Policy in the UK or other commonwealth jurisdictions with similar statutes, it should be revised to fully comply with the law and address our moral concerns in relation to tissue harvest from minor donor siblings.

## Competing interests

The authors declare that they have no competing interests.

## Authors’ contributions

Corresponding and first author CTK is the main researcher, writer and editor responsible for the legal research and ethical analysis. The co-author TGL, was responsible for identifying ethical issues, overseeing the research and giving expert input into the ethical analysis and review. Both authors read and approved the final manuscript.

## Pre-publication history

The pre-publication history for this paper can be accessed here:

http://www.biomedcentral.com/1472-6939/14/43/prepub
